# TLK1B promotes repair of UV-damaged DNA through chromatin remodeling by Asf1

**DOI:** 10.1186/1471-2199-7-37

**Published:** 2006-10-20

**Authors:** Siddhartha P Sen, Arrigo De Benedetti

**Affiliations:** 1 Department of Biochemistry and Molecular Biology and the Feist Weiller Cancer Center, Louisiana State University Health Sciences Center, 1501 Kings Highway, Shreveport, LA 71130-3932, USA

## Abstract

**Background:**

The mammalian protein kinase TLK1 is a homologue of *Tousled*, a gene involved in flower development in *Arabidopsis thaliana*. The function of TLK1 is not well known, although knockout of the gene in *Drosophila*, or expression of a dominant negative mutant in mouse mammary cells causes loss of nuclear divisions and chromosome mis-segregation. TLK1B is a splice variant of TLK1 and it confers radioresistance in a normal mammary mouse cell line possibly due to increased chromatin remodeling capacity, but the mechanism of resistance remains to be fully elucidated.

**Results:**

We now show that TLK1B also affords protection against UV radiation. We find that nuclear extracts isolated from TLK1B-containing mouse cells promote more efficient chromatin assembly than comparable extracts lacking TLK1B. TLK1B-containing extracts are also more efficient in repair of UV-damaged plasmid DNA assembled into nucleosomes. One of the two known substrates of TLK1 (or TLK1B) is the histone chaperone Asf1, and immuno-inactivation experiments suggest that TLK1B increases UV-repair through the action of Asf1 on chromatin assembly/disassembly.

**Conclusion:**

Our studies provide evidence for TLK1B-mediated phosphorylation of Asf1 triggering DNA repair. We suggest that this occurs via Asf1-mediated chromatin assembly at the sites of UV damage.

## Background

*Tousled*-like kinases (TLKs) belong to a family of serine-threonine kinases highly conserved in plants as well as animals [1,2]. *Tousled*-mutants in *Arabidopsis *have abnormal flower development, with defects also in leaf morphology and flowering time [3,4]. Humans have two homologs of *Tousled*: *TLK1 *and *TLK2 *[2]. The exact function of these kinases has not been determined, but they are known to act in a cell-cycle dependent manner. They are maximally active during the S phase, and are also the targets of checkpoint kinases [5]. Specifically, it has been reported that TLK1 is inhibited by ATM-Chk1 by direct phosphorylation at Ser 695 [6]. Knockout of the *Tousled *gene in *Drosophila *and *C. elegans *cause an early arrest in embryonic development [7,8], while expression of a dominant negative mutant in mouse cells causes loss of nuclear divisions and missegregation of chromosomes [9]. The importance of TLK1 in chromosome segregation was also confirmed by a study on *C. elegans *embryos [10]. We recently cloned a cDNA encoding a mammalian *Tousled*-like kinase, through a different scheme, based on the polysomal redistribution of weakly translated transcripts that become preferentially recruited upon overexpression of the translation initiation factor eIF4E [11]. This mRNA is a splice-variant of TLK1 mRNA, and encodes a 60 kDa protein (TLK1B) as compared to the 82 kDa TLK1 protein. TLK1B is translationally regulated by its 5'UTR and both ionizing radiation (IR) and the radiomimetic drug doxorubicin cause an increase in its translation [12]. Unlike TLK1 and TLK2 that are widely expressed in various organs and tissues, TLK1B is expressed at very low levels in normal cells, but is overexpressed in some breast carcinomas [13].

TLK1 has only two known substrates. One is the histone H3, and it is phosphorylated at serine 10 [11]. The other substrate is the histone chaperone Asf1 [1], which, in addition to its role in nucleosome assembly [14-16] and disassembly [17,18], has been shown to have multiple functions. Most importantly, Asf1 has been implicated in DNA repair [14,19,20]. Human Asf1 and CAF-1 (another histone chaperone) have been shown to interact and synergize in a repair-coupled nucleosome assembly pathway [20]. The role of Asf1 in DNA repair has been further confirmed by genetic studies in *S. cerevisiae *[21]. In addition, Asf1 is also associated with checkpoint effectors [22]. In *S. cerevisiae*, it interacts with the unphosphorylated form of the checkpoint kinase Rad53 (ortholog of mammalian Chk2). Upon DNA damage and replication block, Rad53 is phosphorylated and dissociates from this complex, leaving Asf1 free to interact with acetylated histones H3 and H4 [23]. Despite the association of Asf1 and Rad53 in yeast, an Asf1-Chk2 interaction in mammalian cells has not yet been reported. Thus the mechanism of Asf1 regulation might be different, and could be dependent on its phosphorylation status. However, the consequence of Asf1 phosphorylation in mammalian cells remains unknown.

TLK1B overexpression protects mouse mammary cells (MM3-TLK1B) from the genotoxic effects of ionizing radiation (IR) [11]. (We were unable to make a stable cell line that would express the full-length TLK1 protein). Based on this evidence and the fact that ATM and Chk1 are involved in the DNA damage checkpoint, it can be hypothesized that TLKs are involved in some aspect of genome surveillance, particularly chromatin remodeling concurrent with DNA repair. Accordingly, we showed that TLK1B protected cells from IR by facilitating the repair of double-stranded breaks (DSBs) [24]. Using an *in vitro *repair system, we showed that addition of recombinant TLK1B promoted the repair of a linearized plasmid incubated with nuclear extracts, possibly by influencing the assembly of chromatin on the DNA template [24].

In this paper we provide evidence that overexpression of TLK1B also protects cells from the harmful effects of UV-radiation. It is well known that UV-radiation is a potent and ubiquitous carcinogen responsible for the majority of skin cancers [25]. Unlike IR which primarily causes DSBs, UV-induced DNA damage mainly results in the formation of cyclobutane pyrimidine dimers (CPDs) [26]. In humans, CPDs are repaired by a process known as nucleotide-excision repair (NER) [27]. One of the best studied pathways in DNA repair, NER is highly conserved in eukaryotes. There are many differences between prokaryotes and eukaryotes; however, the basic principles are retained. NER is subdivided into two pathways; global genomic repair (GGR) that targets and removes lesions from the whole genome, and transcription-coupled repair (TCR) that preferentially removes lesions from the transcribed strand of expressed genes. It consists of four main steps: 1) Recognition of the damage; 2) Dual excision and removal of the intervening damaged DNA strand; 3) Gap repair synthesis; and 4) Ligation of the nick. In mammalian cells, NER is a complex process involving multiple large protein complexes. Packaging of the eukaryotic DNA into chromatin further complicates the process because in principle, damage accessibility would be dependent on the structural properties of the nucleosomes in and around the site of DNA damage [28-30]. NER is not essential for viability, but defects in the process result in disorders. One example is the genetic disease xeroderma pigmentosum (XP), which is associated with severe light sensitivity and an increased risk of UV-induced skin cancers. These diseases exemplify the importance of UV-induced DNA damage and its repair in humans [31].

Based on clonogenic assays, we first show that TLK1B-overexpressing cells are more resistant to UV radiation. This is further confirmed by an *in vivo *assay showing efficient and faster repair of genomic DNA as well as episomes in intact cells exposed to UV-radiation. Finally we use *in vitro *assays that demonstrate that TLK1B-overexpressing cells promote chromatin remodeling as well as repair of DNA damage, likely by modulating the activity of Asf1.

## Results

### TLK1B protects the cells from UV

**Figure 1:**
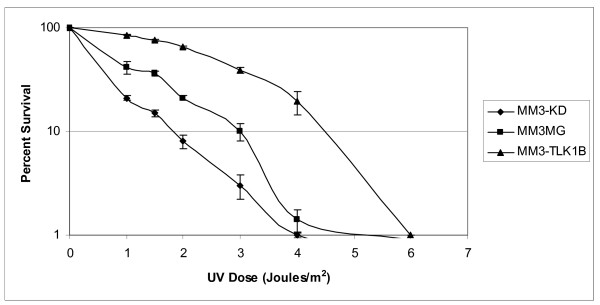
Survival curves after UV irradiation. Percentage survival of MM3MG, MM3-TLK1B and MM3-KD cells after exposure to 1, 1.5, 2, 3, 4 and 6 J/m^2 ^of UV are shown. The average of triplicate experiments is shown. The D_0 _values were 3.5 J/m^2 ^for the MM3-TLK1B cells, 2 J/m^2 ^for the MM3MG cells and 1.2 J/m^2 ^for the MM3-KD cells respectively.

We have previously shown that stable overexpression of TLK1B protects a normal cell line (MM3MG) from IR [11]. Therefore, we investigated if TLK1B could protect these cells from other types of DNA damage, or if it was specific for DSBs. Studies of its role in other types of DNA damage would help us to get a clearer picture of the position of this kinase in the DNA damage response pathway. We tested the effect of UV, which induces formation of pyrimidine dimers, on control cells (MM3MG), cells overexpressing TLK1B (MM3-TLK1B) and cells expressing the kinase dead mutant of TLK1B (MM3-KD) by a clonogenic assay (Figure 1). For making the kinase-dead mutant expressing cells the highly conserved D386 and K389 residues in the ATP-binding pocket of the kinase were replaced with Alanine. *In vitro*, the mutant kinase, expressed as a GST-fusion in bacteria, lost the capacity to phosphorylate itself and histone H3 [9]. The kinase-dead (KD) protein was then expressed from the BK-Shuttle vector as done before for the wt TLK1B. MM3-KD cells may be expected to show increased sensitivity to UV damage, if the KD mutant acts as a dominant negative for TLK1 and TLK2 substrates. The BK-Shuttle vector replicates as an episome with a very consistent copy number in each cell and hence, the transfected population gives homogeneous expression of the transgene, making it unnecessary to select individual G418-resistant clones [32]. This is an important point since the results obtained thereafter cannot be simply attributed to the selection of an aberrant clone.

We found that 21% of the MM3MG cells survive at 2 J/m^2^, 10% of the MM3-KD cells survive, while 63% of the MM3-TLK1B cells survive at the same dose. For the dose of 4 J/m^2 ^also, the survival fraction is considerably higher in MM3-TLK1B cells as compared to MM3MG and MM3-KD cells (19.3% vs. 1.4% vs. 0.7%); (P < 0.01 in both cases). This indicates that overexpression of TLK1B significantly increases the resistance to UV radiation.

### *In vivo *repair of UV damage in MM3MG and MM3-TLK1B cells

**Figure 2:**
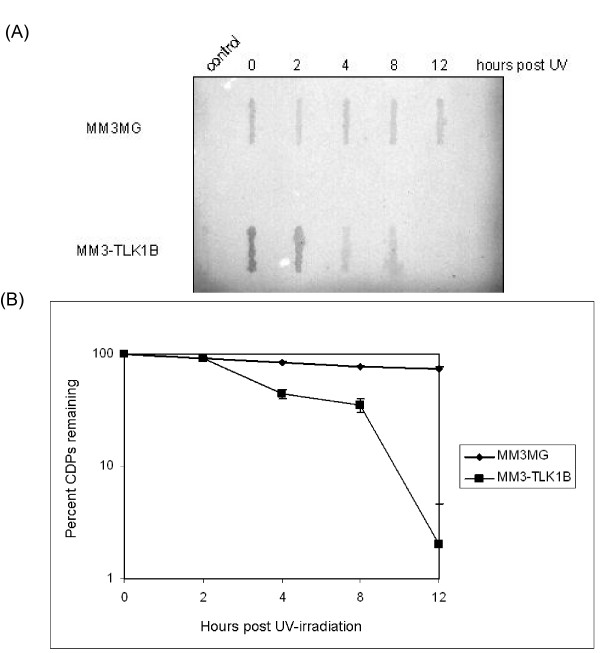
Repair of genomic DNA in MM3MG and MM3-TLK1B cell lines following UV-radiation. (A) A slot blot assay carried out to detect pyrimidine dimmers (CPDs) using a monoclonal antibody. Unirradiated DNA (slot 1) was used as a control. (Representative of three experiments). (B) Graphical representation of the data shown in figure 2A. Average of three experiments is shown. The X-axis denotes the hours of recovery following UV-irradiation, while the Y-axis represents the CPDs remaining in terms of percentage (0 hrs: 100%; No UV control: 0%).

We next wanted to determine the basis behind the increased survival of MM3-TLK1B cells after exposure to UV. Expression of TLK1B did not appreciably alter the transcriptome in MM3MG cell lines (microarray analyses, unpublished data), indicating that the protective effect was likely post-transcriptional. We recently showed that TLK1B promotes the repair of double-stranded breaks (DSBs), most likely by promoting chromatin remodeling at the sites of damage [24]. Therefore we hypothesized that a similar mechanism of more efficient DNA repair might be responsible for the increased survival of MM3-TLK1B cells after exposure to UV radiation. In order to investigate this, the repair of genomic DNA in MM3MG and MM3-TLK1B cells was determined using an immunoblot assay and monoclonal antibodies specific to CPDs, over a time course. As shown in figures 2A and 2B, the MM3-TLK1B cells efficiently repaired almost all the CPDs by 12 hours. On the other hand, MM3MG cells show very poor levels of CPD removal.

**Figure 3:**
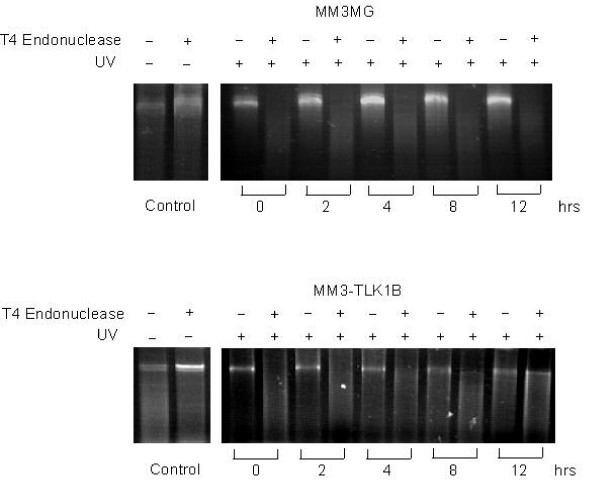
Repair of episomes in intact irradiated cells. MM3MG cells (stably transfected with the empty BK-shuttle vector) and MM3-TLK1B cells were exposed to 5 J/m^2 ^of UV (0 hrs) and allowed to recover for 0, 2, 4, 8 and 12 hours. Episomal vector extracted from both cell lines (control as well as UV-irradiated) at shown time-points was either mock-treated (-) or digested with T4 endonuclease V (+).

Another method was used to compare the repair abilities of the two cell lines. For this purpose, we assessed the repair of episomal vectors in cells over a time course following exposure to UV. MM3MG cells (that were stably transfected with the empty BK-shuttle vector) and the MM3-TLK1B cells were exposed to 5 J/m^2 ^of UV radiation, and allowed to recover for 0 to12 hours (Fig. 3). Episomes were extracted at various time points (0, 2, 4, 8 and 12 hours) and were either mock-treated or digested with T4 endonuclease V enzyme, which specifically cuts DNA at CPD-sites. Since the episomes are damaged on both strands at multiple sites, digestion with T4 endonuclease results in extensive cleavage of the plasmid DNA, whereas in unirradiated cells T4 endonuclease leaves the plasmids intact (see control lanes). Figure 3 shows the repair ability of MM3MG and MM3-TLK1B cell lines by this method. On inspection of the two gels it was seen that the majority of low molecular weight episomal DNA was recovered into repaired high molecular weight DNA in the MM3-TLK1B cells between 8 and 12 hours, while the T4 endonuclease treated episomal DNA still appeared as a low molecular weight smear in the MM3MG cells. These two experiments strongly suggest that the MM3-TLK1B cells repaired DNA faster than the control MM3MG cells.

### The phosphorylation of H3 recovers faster in TLK1B-expressing cells

**Figure 4:**
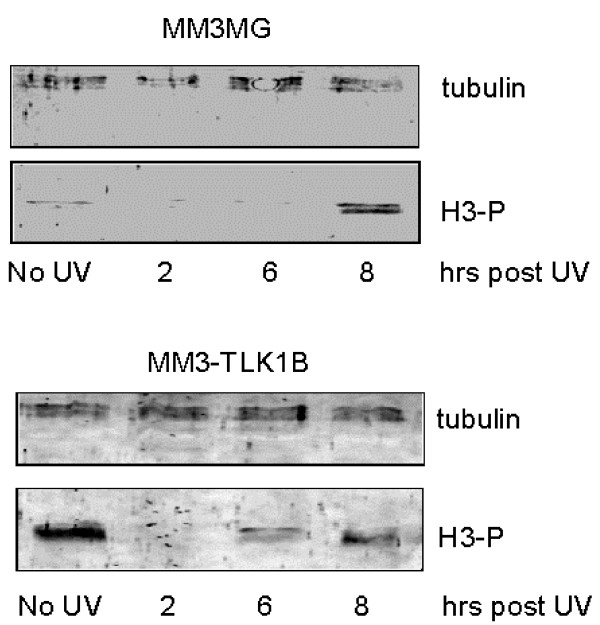
Phosphorylation of H3-Ser 10 after UV-irradiation. MM3MG and MM3-TLK1B cells were grown to 80% confluence prior to UV-radiation (5 J/m^2^). Cells were harvested at different times (0, 2, 6 and 8 hrs) after irradiation and lysed in RIPA buffer. Equal amount of protein of each sample was loaed on a 15% SDS-PAGE gel and electrophoresed. Blots were probed with phospho-Histone H3 (Ser 10) antibody. Equal loading of proteins was confirmed by blotting for tubulin. These blots are representative of two separate experiments.

Genotoxins cause a temporary, ATM-dependent, inactivation of TLK1 [6] that can best be monitored through a loss of phosphorylation of histone H3-S10 [9]. We set out to test whether UV would also be a treatment that caused dephosphorylation followed by re-phosphorylation of H3 when the cells are allowed to recover post treatment. Indeed, there was a dramatic loss of H3P-S10 following UV damage, which persisted for 8 hours in the MM3MG cells, but only for 6 hours in the cells expressing TLK1B (Figure 4). Therefore, if the phosphorylation of H3 can be taken as an indication of repair activity in UV-damaged cells, then there is a much faster recovery/repair in cells expressing TLK1B. The phosphorylation of H3 was taken as an indicator of TLK1 activity/inactivation, since no change in mobility was observed for the other substrate of TLK1, Asf1 (data not shown) following UV. We should stress that the change in H3 phosphorylation is most likely through an ATM (or more likely ATR in the case of UV)-mediated inhibition of TLK1 through Chk1 [6] and not a change in the cell cycle (like a mitotic arrest) since the loss of H3 phosphorylation after UV was immediate and reversible.

### *In vitro *assays to study the repair of UV-induced DNA damage

**Figure 5:**
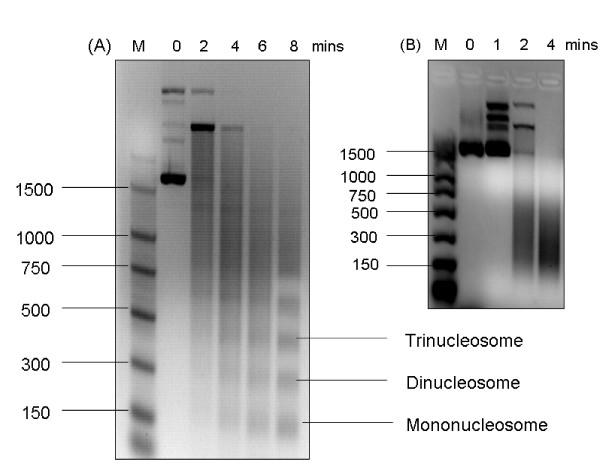
(A) MNase digestion of pBluescript assembled into nucleosomes. Lane 1:Marker; Lanes 2–6: MNase treatment for 0, 2, 4 and 8 minutes respectively. (B) MNase digestion of naked plasmid.

**Figure 6:**
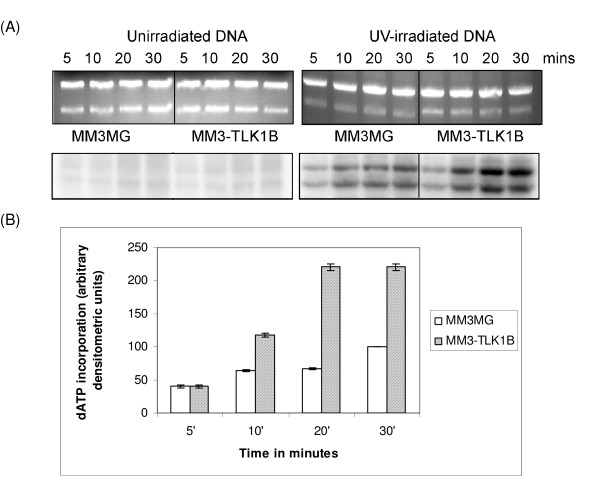
(A) In vitro DNA repair assay on nucleosomal template showing incorporation of [α]^32^P-dATP over a time course by MM3MG and MM3-TLK1B extracts. The left panel shows unirradiated DNA while the right panel shows UV-treated plasmid DNA. The numbers at the top of the gel indicate the time for which the plasmid was incubated in the extract, ranging from 5 to 30 minutes. The figure shown is a representative of five independent experiments. (B) Graphical representation of the data shown in figure 6(A). The maximum repair in the MM3MG (control) cell line has been set at 100% (30 minute time point) and the standard-deviations from three independent experiments have been used for the error bars.

We elected to use an *in vitro *reconstituted system to study the role of TLK1B in the repair process. For these assays we used UV-damaged Bluescript plasmid which was preassembled into a chromatin template. Nucleosome assembly on supercoiled plasmid was done by the deposition of histones using salt dialysis, and efficient assembly was confirmed by observation of a regularly protected DNA ladder on digestion with micrococcal nuclease (MNase), as shown in Figure 5A. As a control, Bluescript plasmid not assembled into nucleosomes was digested completely by MNase, resulting in a smear (Figure 5B). Repair on pre-assembled nucleosomal templates was measured by the incorporation of radiolabeled nucleotides into plasmid DNA during repair synthesis catalyzed by nuclear extracts of MM3MG and MM3-TLK1B cell lines. Unirradiated plasmid DNA was used as a negative control, and only minimal background incorporation of label was observed in this mock NER assay. Figures 6A and 6B show that only reactions with irradiated plasmid DNA show incorporation of radiolabeled nucleotide, which suggests that the label is incorporated at sites of repair of UV damage, and the fact that more than twice as much repair is seen in the reactions containing extracts from MM3-TLK1B cells indicates that the presence of TLK1B stimulates repair. Similar results were obtained when plasmids not preassembled into nucleosomes were used for the repair assay (data not shown), but we should point out that this is likely due to the fact that the plasmid was getting assembled into chromatin by extract-mediated chaperones and endogenous histones (data not shown).

### TLK1B stimulates chromatin assembly *in vitro*

**Figure 7:**
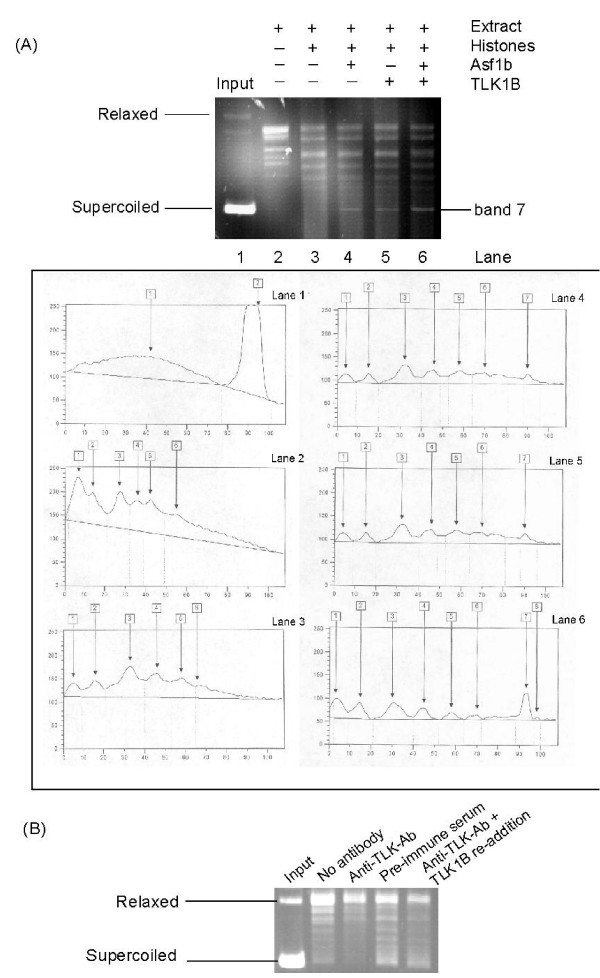
A Chromatin assembly (supercoiling assay) of bluescript plasmid with MM3MG extract supplemented with Asf1 and/or TLK1B. Input: Fully supercoiled plasmid prior to incubation in nuclear extract. Plasmid was assembled into chromatin by incubation (for 1 hour at 37°C) with extract, energy mix and the specified components indicated. The figure is a representative of three separate experiments. The densitometric profile for each condition is shown. Note the increase in intensity of band 7 in lane 6. B Chromatin assembly (supercoiling assay) of bluescript plasmid in the presence of TLK1-antiserum (MM3MG extracts). Lane 1: Input plasmid; Lane 2: Plasmid + extract; Lane 3: Plasmid + extract + TLK1-antiserum; Lane 4: Plasmid + extract + pre-immune serum. Lane 5: Plasmid + extract + TLK1-antiserum + recombinant TLK1B re-addition. The assay shown is a representative of two independent experiments. (MM3MG and MM3-TLK1B extracts gave similar results).

From the results obtained in Figure 6, it can be inferred that there is more efficient DNA repair of the plasmids incubated with MM3-TLK1B extracts. The only currently known substrates of TLK1 (or TLK1B) are histone H3 [11] and Asf1 [1], which suggest that perhaps chromatin remodeling is involved in the more efficient repair process. The Bluescript DNA was fully competent as a substrate for repair synthesis, but the previous assay yielded little evidence about the chromatin remodeling capacity of the MM3MG and MM3-TLK1B extracts. Therefore, we investigated the ability of TLK1B to enhance the assembly of chromatin by an *in vitro *plasmid supercoiling assay (Figure 7A). In this assay, the Bluescript plasmid (nearly 100% supercoiled, *input*, lane 1) was used as a template for the deposition of core histones in the presence of MM3MG nuclear extract and an energy mix. In the absence of exogenous histones, the extract causes the bacterially supercoiled form to convert to mostly the relaxed form due to endogenous topoisomerases (lane 2). However, after incubation in the presence of histones, the plasmid migrates as a series of discrete supercoiled forms due to the formation of nucleosomes, which decrease the linking number by one integer (i.e., one negative supercoil) per nucleosome. The addition of recombinant TLK1B stimulated the formation of the more highly supercoiled forms, particularly a form that comigrates with the bacterially supercoiled plasmid (lane 5, band 7), and thus maybe fully assembled into nucleosomes, i.e. one nucleosome per 200 bp, mimicking the superhelical density (-0.05) of the bacterially propagated plasmid. Asf1 has been shown to stimulate chromatin assembly *in vitro *[16]. When recombinant Asf1b protein was added to the *in vitro *reaction (lane 4), it also increased the supercoiling. Strikingly, maximum supercoiling was seen in lane 6 when both Asf1 and TLK1B were included. This increase in supercoiling is possibly due to the interaction of TLK1B with Asf1, wherein TLK1B increases the chromatin assembly activity of Asf1. The results of the supercoiling assay are further confirmed by the densitometric analysis of each lane (Figure 7A). In comparison to lanes 2 and 3, lanes 4, 5 and 6 show an additional band (band 7), which, as discussed above, may represent the fully supercoiled topoisomer that runs like the most supercoiled band in lane 1. However, the intensity of this band is 5–6 fold higher in lane 6 (when both Asf1b and TLK1B are included) as compared to lanes 4 and 5.

The importance of TLK1 (or TLK1B) in the chromatin assembly is further demonstrated when an antibody that inactivates TLK1 is added to the *in vitro *chromatin assembly reaction prior to the addition of the plasmid. As is seen in Figure 7B, addition of anti-TLK1 antibody decreased the supercoiling (lane 3), while no appreciable effect was seen when a non-specific antibody was used (lane 4, pre-immune serum). At the same time, addition of recombinant TLK1B protein to the TLK-depleted extract could restore the supercoiling activity considerably, indicating that the recombinant protein could complement the depletion by the antibody (lane 5). Both extracts showed very similar results in this case.

**Figure 8:**
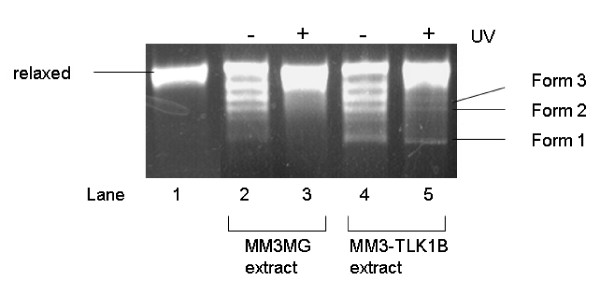
Chromatin assembly (supercoiling assay) of undamaged (lanes 2 and 4) and UV-damaged (lanes 3 and 5) pBluescript by MM3MG (lanes 2 and 3) and MM3-TLK1B (lanes 4 and 5) extracts. Note the presence of more supercoiled topomers (Forms 1,2 and 3) in lane 5 as compared to lane 3.

To analyze the supercoiling activity of the two extracts during repair synthesis (which is dependent on CAF-1) a similar assay was performed, but using completely relaxed UV-damaged plasmid as a substrate. In such an assay, NER nicking of the plasmid at CPDs will tend to maintain the plasmid in a relaxed form, whereas at the same time extract-mediated chromatin assembly will decrease the superhelicity of the plasmid generating faster migrating forms. As shown in Figure 8, completely relaxed UV-damaged plasmid was incubated with either MM3MG or MM3-TLK1B extract. As a control, undamaged plasmid was used for comparison. From the figure, it is clear that the MM3-TLK1B extract induces more supercoiling of the UV-damaged plasmid, while the plasmid remains in its relaxed form when incubated with the MM3-MG extract (compare lanes 3 and 5). This confirmed that the TLK1B extract has better chromatin remodeling activity for UV-damaged plasmids.

### Role of TLK1-Asf1 relationship in DNA repair

**Figure 9:**
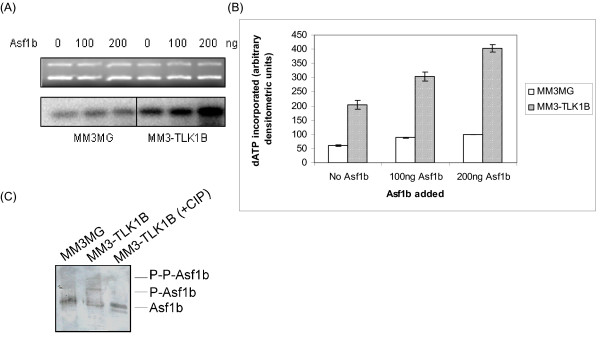
(A) In vitro repair assay using increasing amounts of recombinant Asf1b. Lanes 1–3: Repair by MM3MG extracts; Lanes 4–6: Repair by MM3-TLK1B extracts. Asf1b increases from 0 to 200 ng as shown on the top. (B) Graphical representation of the data in figure 9A. The maximum repair in the MM3MG (control) cell line has been set at 100%(Lane 3, 200 ng Asf1b) and the standard-deviations from three independent experiments have been used for the error bars. (C) Western blot of MM3MG and MM3-TLK1B extracts using α-Asf1b antibody.

The role of Asf1 in DNA repair and maintenance of genomic integrity has been well documented. Cells lacking CAF-1 and RCAF (a complex of Asf1, and acetylated histones H3 and H4) [14] are hypersensitive to DNA-damaging agents, such as camptothecin, suggesting a possible defect in double-strand break (DSB) repair [19]. In budding yeast lacking Asf1, there is activation of the DNA damage checkpoint [21]. Asf1 has also been shown to act synergistically with CAF-1 to assemble nucleosomes during nucleotide excision repair *in vitro *[20]. It is possible that increased repair synthesis by the MM3-TLK1B extracts can be attributed to more efficient chromatin remodeling coupled DNA repair because of a more active Asf1. If TLK1B increases the activity of Asf1, then addition of Asf1 should give a comparable result as addition of TLK1B. In order to determine this, the *in vitro *repair assay was repeated on the UV-damaged nucleosomal Bluescript template, but with increasing amounts of recombinant Asf1b protein. Since the effect of MM3-TLK1B on repair synthesis was evident as early as 10 minutes (Figure 5, compare lanes 2 and 6 of the right panel), this assay was performed for 10 minutes. The results are shown in Figure 9A. As seen before, the repair synthesis by the MM3-TLK1B extracts was several fold higher than that by the MM3MG extracts (compare lanes 1 and 5). However with increase in the amount of Asf1b protein (100 and 200 ng), there was a considerably higher amount of dATP incorporation by the MM3MG cell extracts (compare lanes 1, 2 and 3). Addition of recombinant Asf1b protein also increased the repair synthesis in case of the plasmid treated with the MM3-TLK1B extract. In fact, addition of recombinant Asf1b resulted in a similar increase in dATP incorporation in both the cases (1.5 times increase with 100 ng and 2 times increase with 200 ng). A graphical representation with three repeats of this experiment is shown in Figure 9B.

When a western blot for Asf1b was done on whole cell extracts of both the cell lines, we clearly detected higher migrating forms of the protein in the MM3-TLK1B extracts as compared to the MM3MG extracts (Figure 9C, compare lanes 1 and 2), indicating that it is more phosphorylated. That the slower migrating forms were due to phosphorylation was confirmed when treatment with calf-intestinal alkaline phosphatase (lane 3) caused the Asf1b in the TLK1B cells to migrate like the Asf1b in the MM3MG cells. The most direct interpretation of these results is that TLK1B extracts have increased activity of Asf1 due to phosphorylation.

**Figure 10:**
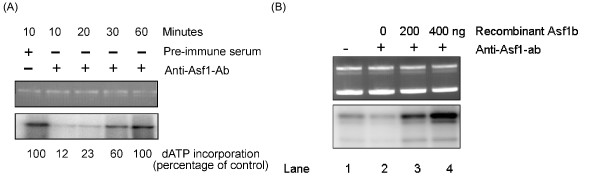
(A)*In vitro *repair assay in the absence (lane 1) or presence (lanes 2–5) of Asf1-antiserum (MM3MG extracts). The numbers below each lane indicate the amount of radiolabel incorporated as percentage of control (lane 1), while the numbers at the top indicate the period of plasmid incubation in the presence of MM3MG nuclear extract. MM3MG and MM3-TLK1B extracts gave similar results. (Representative of two separate experiments). (B) In vitro repair assay in the presence of Asf1-antiserum (lane 2) followed by complementation with recombinant Asf1b protein (lanes 3 and 4).

The role of Asf1 was further demonstrated by the results shown in Figure 10A. In this case the repair assay was done in the same way as described before (incubating the UV-damaged nucleosomal DNA template with nuclear extracts, energy mix and [α]^32^P-dATP for 10–60 minutes), but Asf1-antiserum was added to the reaction mix, prior to addition of the plasmid (lanes 2–5). That the Asf1-antibodies bind the native proteins was determined by immunoprecipitation (data not shown). As a control, the reaction was also done in the presence of a non-specific antibody (pre-immune serum, lane 1). There was a significant decrease in the repair synthesis in the presence of Asf1 antiserum (compare lane 1 with lanes 2, 3 and 4). Only when the reaction was allowed to continue for a longer time (60 minutes, lane 5), did the repair synthesis reach levels similar to that seen in lane1. Similar results were obtained with both the MM3MG and MM3-TLK1B extracts. This shows that Asf1 is important for the DNA repair process, even in the presence of TLK1B in the extract. Interestingly, Asf-1 depletion did not show any appreciable effect in case of repair assays that were done on non-chromatin UV-damaged templates (data not shown). This indicates that Asf1 only affects repair during assembly/disassembly of chromatin, and it is not intrinsically increasing the overall repair reaction on UV damaged templates.

The effect of the Asf1-antiserum could also be complemented by adding back recombinant Asf1b protein, indicating that it was specifically interfering with the Asf1 function during the repair process (Figure 10B).

### Role of TLK1B in the DNA repair process

**Figure 11:**
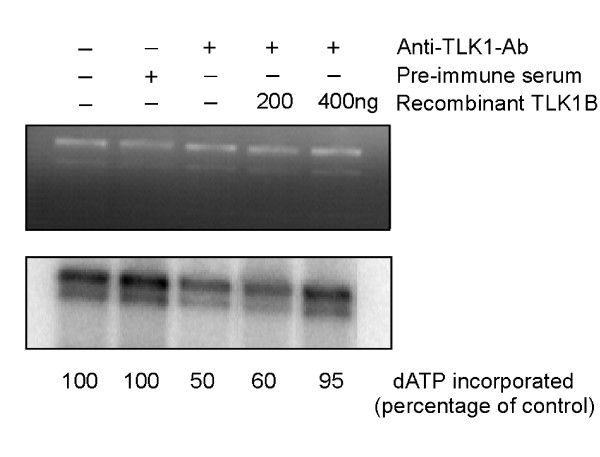
*In vitro *repair assay in the presence of TLK1-antiserum (lanes 3, 4 and 5), followed by complementation with recombinant TLK1B protein (lanes 4 and 5). Numbers at the bottom indicate the amount of radiolabel incorporated as percentage of control (lane 1). MM3MG and MM3-TLK1B extracts gave similar results. (Representative of two separate experiments).

Next, we tested the role of TLK1B itself using the *in vitro *assays (Figure 11). The *in vitro *repair assay was repeated as before, however, in the presence (lane 3) or absence (lane 1) of TLK-1 antiserum. As a control, a non-specific antibody (pre-immune serum, lane 2) was also used. We saw that the repair synthesis diminished by half (lane 3) as compared to the control extracts (lanes 1 and 2), as indicated by the amount of radiolabel incorporated. However, when increasing amounts of recombinant TLK1B were added to the *in vitro *reaction, it could restore repair synthesis in the TLK-depleted extracts. Here too, similar results were seen in case of both MM3MG and MM3-TLK1B extracts.

## Discussion

TLK1 and TLK2 were originally cloned during a PCR-based search for human kinases [2]. We identified a splice variant of TLK1 (TLK1B) using a completely different approach, based on differential redistribution of transcripts on the polysomes of cells overexpressing the translation factor eIF4E [11]. It stands to reason that the phenotypical changes induced by eIF4E overexpression, which include transformation in several cell lines, would be mediated by a change in the recruitment of mRNAs on the polysomes and the corresponding increase in protein expression. One of the known changes induced by eIF4E is an increase in resistance to genotoxins and resistance to apoptosis [33]. We then set out to uncover that TLK1B was conferring resistance to IR when overexpressed by itself even in the absence of eIF4E. In this report, we set out to study whether the effects of another genotoxin (UV) could also be attenuated by overexpressing TLK1B, and if so, the possible mechanism for UV resistance, which we postulate is mediated through upregulation of Asf1 activity.

In a previous publication we had shown that TLK1B overexpression induced resistance to IR and this was due to more efficient chromatin remodeling coupled to DNA repair [24]. To assess whether TLK1B protected cells from UV-induced DNA damage, we carried out clonogenic assays which showed a highly significant increase in survival, while the converse was observed in a cell line expressing a dominant negative mutant of TLK1B. This indicated that TLK1B might have a more general role in repair of DNA damage than just protection from IR. Indeed, we subsequently found that the repair of genomic DNA following exposure to UV was faster and more efficient in MM3-TLK1B cells as compared to MM3MG cells. In addition, repair of UV damaged episomes in the TLK1B overexpressing cells was also significantly faster and more complete than in control cells. We should point out that the episomes are packed in regular chromatin in mammalian cells and thus mimic repair of genomic DNA [34]. So episomal repair can be taken as an indicator of the repair that occurs on genomic DNA, which is generally more difficult to assess *in vivo*. To assess the mechanism of TLK1B-induced resistance to UV radiation, we resorted to *in vitro *assays for a more direct assessment. We assayed UV-damaged plasmids that had been packaged into chromatin for two main reasons. First, chromatin is the authentic substrate of NER *in vivo*, and second, the effect of TLK1B was expected to be most likely due to a change in chromatin remodeling capacity.

The role of chromatin remodeling in cellular processes like transcription is well established, but only recently is it being studied in the context of DNA repair. Several lines of evidence clearly indicate that the presence of nucleosomes on damaged DNA severely hampers access of the DNA repair machinery [35,36]. At the same time, chromatin remodeling facilitates the repair process. Recombinant ACF, an ATP-dependent chromatin remodeling factor, was found to facilitate the dual incision on a dinucleosomal template [37]. The Cockayne Syndrome-B (CSB) protein involved in transcription-coupled repair has homology to the SWI/SNF2 family of chromatin remodeling complexes and has chromatin remodeling activity [38]. Although not a classic 'chromatin remodeler', the histone chaperone Asf1 plays an important role in several processes that are very much dependent on the chromatin structure. In addition to its well-studied role in chromatin assembly, yeast Asf1 has also been shown to be important in silencing, transcription, DNA replication and normal cell cycle progression [22,23,39,40]. Most importantly though, Asf1 has been implicated in the maintenance of genomic integrity [39]. Yeast cells deleted for *ASF1 *are highly sensitive to DNA-damaging agents [14] and Asf1 mutants have an increased rate of genomic instability [40]. In mammalian cells, Asf1 is phosphorylated by TLK1. In fact, the only two known substrates of TLK1 thus far are histone H3 and Asf1. Also, Asf1 phosphorylation in the S-phase has been shown to correlate with TLK1 activity [1]. This suggests that TLK1 and TLK1B have a function in chromatin remodeling, particularly TLK1B, which may have a function in remodeling during the DNA damage repair pathway by NER.

We have shown in this study that the extract containing TLK1B was far more active, both in repair of UV-damaged chromatin templates, as well as plasmid supercoiling. The role of TLK1/TLK1B was further confirmed by immuno-inactivation/complementation experiments. TLK1B's effect is almost certainly due to modulation of Asf1 activity, as addition of Asf1 alone to control extracts could restore repair activity to the level seen with TLK1B-containing extract, while Asf1 depletion resulted in a considerable decrease in the repair synthesis. It should be noted that depletion of Asf1 resulted in a decrease in the repair synthesis only in the earlier time points, but by one hour, the final extent of repair was equivalent to that seen with extract incubated with a non-specific antibody (Figure 8, lanes 1–5). A recent study showed that Asf1 increased the rate of histone eviction at the PHO5 promoter in *S. cerevisiae *[41]. In the absence of Asf1 histone eviction was delayed but the final outcome of the chromatin transition was not affected. Our results indicate a similar finding. Asf1 depletion probably results in inefficient disassembly of chromatin at the sites of DNA damage, resulting in a delay in repair. But at later time points (probably because of the action of other chromatin remodeling factors), there is chromatin remodeling followed by repair of the damage.

## Conclusion

In conclusion, our results show that TLK1B enhances the repair of UV-damaged DNA in the context of chromatin, and most likely does so through the action of Asf1, though other possible substrates cannot be excluded. The future challenge will be to determine if there are other substrates of this kinase, and if so, whether they have any role in DNA damage-response and repair.

## Methods

### Cell lines and tissue culture

Normal breast epithelial cells, MM3MG, transfected or not with TLK1B were cultured as described in Li *et al*. [11]. MM3MG cells expressing the kinase dead mutant of TLK1B (MM3-KD) were described in Sunavala-Dossabhoy *et al*. [9].

### Clonogenic assay

For the clonogenic assay, MM3MG cells, cells overexpressing TLK1B (MM3MG-TLK1B) and cells expressing the kinase dead mutant of TLK1B (MM3-KD) were harvested with PBS/EDTA and adjusted to 10,000 cells/dish in PBS. Cells were then treated with the mentioned doses of UV using a germicidal UV lamp (254 nm) kept at a fixed distance from the dishes. The fluence rate was measured by a UV-meter (UVP Inc., Upland, CA). For each UV-dose level (0 to 6 J/m^2^), aliquots of serially diluted cells (100–5000) were plated on 6-well plates in triplicate. After a period of 10 days of incubation, the wells were rinsed with PBS, stained with crystal violet, and the colonies counted. The experiment was repeated thrice, and the results were expressed as the fraction of surviving cells compared to the number of colonies formed in the non-irradiated samples (plating efficiency).

### Analysis of genomic repair by slot blots

80% confluent MM3MG and MM3-TLK1B cultures were irradiated with 5 J/m^2 ^of UV. The cells were then collected at various times, and the genomic DNA was isolated using the Wizard SV kit (Promega, Madison, WI) as described by the manufacturer. 200 ng of DNA from the UV-irradiated as well as mock-irradiated cells was spotted onto Immobilon-N (Millipore, MA) using a slot blot apparatus. The filter was then baked for 2 hours at 80°C. Quantification of the CPDs was carried out using a mouse monoclonal antibody for CPDs (MBL International, MA), by densitometry using the ImageQuant software (Molecular Dynamics, Sunnyvale, CA). Antibody binding was determined using the Opti4CN reagent (Biorad, Hercules, CA).

### Analysis of episomal repair by T4 Endonuclease V

MM3MG cells (transfected with the empty BK-shuttle vector) and MM3-TLK1B cells grown to 75% confluence were either sham treated (sample used as non-irradiated control) or UV-irradiated with a dose of 5 J/m^2^. One dish of each cell line was immediately harvested (0 hr time point). The other flasks were re-incubated to allow DNA repair for 12 hours and the cells were harvested at 2, 4, 8 and 12 hour time points respectively. Episomes were extracted by alkaline lysis. Purified DNA (2 μg) was then either mock treated or digested with T4 endonuclease V (Epicentre, Madison, WI) according to the manufacturer's instructions. T4 endonuclease V specifically incises the unrepaired UV-induced CPDs in DNA. The cleavage products were then separated on a 0.8% agarose gel for one and a half hours at 4°C, and stained with ethidium bromide.

### Western blot for H3-Ser10-phosphorylation

The anti-histone H3 phosphorylated at Ser-10 antibody was purchased from Upstate Cell signaling (Lake Placid, NY). For Western blots, 25 μg of protein of each sample was separated on a 15% SDS/PAGE gel. The proteins were transferred to nitrocellulose, incubated with primary antiserum for 1 hour followed by secondary antiserum for 1 hour (1:1000 dilution each). Finally, the membranes were washed and developed using the Opti-4CN reagent (Biorad, Hercules, CA).

### Assembly of Bluescript plasmid into a nucleosomal template

Native core histones were purified from HeLa cells by hydroxylapatite based chromatography according to the protocol described by Simon and Felsenfeld [42]. These purified core histones were then used for assembling nucleosomes. The plasmid Bluescript (pBS) was first damaged with 300 J/m^2 ^of UV (a fluence of 100 J/m^2 ^induces roughly 1 pyrimidine dimer photoproduct in 1000 bp) (Wood *et al*.) [43], following which it was assembled into a nucleosomal template by salt dialysis according to the protocol described by Jeong *et al*. [44]. Briefly, supercoiled pBS was incubated with the purified core histones (in a 2 M NaCl containing buffer) and then subjected to three sequential dialysis steps, in which the salt concentration was gradually decreased. Chromatin assembly was then confirmed by doing a micrococcal nuclease (MNase) digestion and processed for electrophoresis on a 1.5% agarose gel.

### *In vitro *DNA repair assays

Nuclear extract from MM3MG and MM3-TLK1B cells was prepared as described by Gaymes *et al*. [45]. Repair assay was done according to the protocol described by Mello et al. [20], but with some modifications. Reactions contained the damaged plasmid assembled into nucleosomes, MM3MG or MM3-TLK1B nuclear extract, 5 mM MgCl_2_, 40 mM Hepes, pH7.8, 0.5 mM DTT, 4 mM ATP, 20 μM dNTPs, 4 mM phosphocreatine and [α]^32^P-dATP. After incubation at 37°C for varying time periods, the reaction was stopped by transferring on to ice, the plasmid extracted using the Gene Clean kit (Bio 101, Vista, CA) and run on an agarose gel. The gel was then dried and the amount of [α]^32^P-dATP incorporated determined by phosphor-imager analysis (Molecular Dynamics, Sunnyvale, CA) using the ImageQuant software. Undamaged plasmid incorporated into nucleosomal template was used as a negative control.

### Antibodies and recombinant proteins used

The rabbit TLK1-antiserum used in the *in vitro *repair and supercoiling assays was prepared in our lab (Li *et al*.) [11]. Antiserum to Asf1a and Asf1b proteins were obtained from the CIM Antibody Core at Arizona State University. TLK1B and Asf1b proteins that were used in our experiments were expressed in *E. coli *as GST-fusion proteins. The pGEX-CIA plasmid that encodes the full length GST-Asf1b protein was a kind gift from Dr. Masami Horikoshi, Institute of Molecular and Cellular Biosciences, The University of Tokyo, Japan. For figures 9, 10 and 11, the amount of [α]^32^P-dATP incorporated was determined as described in the *in vitro *repair assays, by phosphor-imager analysis (Molecular Dynamics, Sunnyvale, CA) using the ImageQuant software.

### Assay of chromatin assembly

Nucleosome assembly was carried out on 2 μg of Bluescript plasmid. Reactions contained 15 μg of MM3MG cell extract (which already contains sufficient amounts of topoisomerases), 5 mM MgCl2, 40 mM Hepes, pH 7.8, 0.5 mM DTT, 4 mM ATP, 20 μM dNTPs, 4 mM phosphocreatine, 2 u of creatine phosphokinase, and additional purified proteins (200 ng TLK1B, 100 ng Asf1b and 2 μg supplemental HeLa histones). The reactions were incubated at 37°C for 1 hr. The plasmid was re-extracted with the GeneClean kit (Bio 101, Vista, CA), separated on an agarose gel and subsequently stained with ethidium bromide.

## Authors' contributions

SS prepared figures 1 to 11. SS and ADB wrote the paper. Both authors read and approved the final manuscript.
